# Prevalence of mind and body exercises (MBE) in relation to demographics, self-rated health, and purchases of prescribed psychotropic drugs and analgesics

**DOI:** 10.1371/journal.pone.0184635

**Published:** 2017-09-15

**Authors:** Lina Rådmark, Linda L. Magnusson Hanson, Eva Bojner Horwitz, Walter Osika

**Affiliations:** 1 Department of Clinical Neuroscience, Karolinska Institutet, Stockholm, Sweden; 2 Center for Social Sustainability, Department of Neurobiology, Care Sciences and Society, Karolinska Institutet, Stockholm, Sweden; 3 Stress Research Institute, Stockholm University, Stockholm, Sweden; 4 Department of Public Health, Uppsala University, Uppsala, Sweden; 5 Stress Clinic, Stockholm, Sweden; Universidade Federal do Rio de Janeiro, BRAZIL

## Abstract

This study aims to identify any differences regarding gender, age, socioeconomic status (SES), self-rated health, perceived stress and the purchase of prescribed drugs among people who practice mind and body exercises (MBE) extensively compared to people who do not. Methods: The study includes 3,913 men and 4,803 women aged 20–72 who participated in the Swedish Longitudinal Occupational Survey of Health (SLOSH). The respondents were divided into three groups depending on frequency of MBE practice (never/seldom/often). Measures regarding MBE practice, health behaviors, self-rated health, and illnesses were drawn from the SLOSH questionnaire, while more objective measures of socioeconomic status and education were derived from registry data. In addition, data on purchases of prescription drugs for all respondents were included in the study. These data were obtained from the Swedish Prescribed Drug Register, which contains information about prescription drugs dispensed at Swedish pharmacies. Separate analyses were performed for mental MBE (mindfulness, meditation, relaxation techniques) and physical MBE (yoga, Tai Chi, Qi Gong), respectively. Results: A high intensity MBE practice is cross-sectionally related to poor self-assessed health (sleeping problems, pain, depressive symptoms, mental disorders), high levels of stress, and high levels of purchases of psychotropic drugs and analgesics. These cross-sectional relationships are generally stronger for mental MBE than for bodily-directed MBE. More women than men are practicing MBE on a regular basis, and physically active people participate to a greater extent in MBE compared with the physically inactive. Conclusion: Overall, the study shows that frequent participation in mind and body exercises is associated with high levels of purchases of psychotropic drugs and analgesics as well as with poor self-assessed health and high levels of stress. However, since this is a cross-sectional study, it is impossible to establish cause and effect, and to further investigate the associations found; longitudinal studies that can account for temporality between covariates and MBE use are needed.

## Introduction

The use of mind and body exercises (MBE) such as mindfulness, meditation, relaxation techniques, yoga, tai chi and qi gong in order to improve general health, reduce various kinds of pain, decrease stress and stress-related health problems (e.g., anxiety, depression, pain, sleep problems), is common in the Western world [1, 2]. Furthermore, there seems to be an increase in the total use of MBE. A recent report from the National Center for Health Statistics (USA) states, for example, an almost twofold increase (5.1% to 9.5%) of yoga practice among adults in the USA during the period of 2002–2012 [3]. MBE seem to be used both as preventive measures and for treatment [1, 2]. The techniques have been described as being directed towards improvements in emotional regulation, body awareness and relaxation, and have shown beneficial effects in adults with stress-related symptoms, including increased subjective well-being, improved behavioral regulation, reduced psychological symptoms and emotional reactivity [4, 5].

Some MBE techniques have been researched more extensively, and a recent meta-analysis by Goyal et al. (JAMA 2014) shows that a mindfulness meditation program provides a significant improvement in anxiety, depression and pain [2]. This is consistent with findings in previous meta-analysis and review articles that indicate a small to medium effect of meditation and mindfulness on the reduction of, for example, stress, anxiety and depression [6–9]. Some randomized controlled studies show significant effects of participating in mindfulness cognitive behavior therapy in order to prevent relapse after depression [9, 10], and a recent randomized controlled trial shows that mindfulness group therapy in primary care patients with depression, anxiety, and stress is not inferior to individual-based therapy, including cognitive behavioral therapy [11]. However, high-quality controlled trials are still sparse [12].

Although body exercises are not quite as well researched as mindfulness-based therapy, there are several meta-analysis and review articles showing positive associations between practicing yoga and lower levels of depression, anxiety and stress [13–18]. As for the association between practicing yoga and reduction of pain, there is stronger evidence with, for example, chronic or subacute low back pain [19–21]. Regarding tai chi, the research is less extensive, however there are indications that the technique is associated with improvements in psychological well-being [22].

There are currently no evidence-based golden standard treatments for stress-related problems, and no prescribed drugs are directed against stress per se [23, 24]. Still, many patients receive pharmacological treatment such as antidepressants, anxiolytics, hypnotics and analgesics for symptoms more or less associated with stress [25]. The prescription of these drugs is increasing in Sweden [26]. For example, the use of antidepressants in Sweden increased more than fivefold during the period of 1995–2011 [27–29]. Though pharmacotherapy is generally the first line of treatment for depression and anxiety, there is growing evidence that it has limitations, related to both low or no effect, non-adherence, and over and under prescription. Many patients continue to be symptomatic despite seemingly adequate treatment, including combinations of drugs [30, 31]. Furthermore, a recent study [32] shows that many patients have a negative perception of their antidepressant treatment.

There is some evidence that patients with, for example, depression and anxiety are more likely than healthy persons to often use activities such as yoga in addition to prescribed drugs [33–35]. However, there is still a lack of knowledge regarding the prevalence of MBE in the general population and its relation to self-assessed health and proxy markers of disease, such as with prescription of different drugs.

By analyzing data from an established epidemiological cohort including over 18,000 participants, combined with prescription data from the Swedish Prescribed Drug Register, this study aimed to identify any differences regarding gender, age, socioeconomic status (SES), self-rated health, perceived stress, and the purchase of prescribed drugs, among people who practice MBE extensively compared to people who do not practice MBE.

## Methods

### Participants

The study population consisted of participants of the Swedish Longitudinal Occupational Survey of Health (SLOSH)—a nationally representative longitudinal survey of social situation, work life and health that is conducted biennially. The SLOSH sample was drawn from the Swedish Work Environment Survey (SWES), which in turn is a subsample of gainfully employed people aged 16–64 years from the Labour Force Survey. The first wave of SLOSH in 2006 (n = 5,985; response rate 65.0%) was based on the SWES 2003, and for the second wave conducted in 2008 (n = 11,441; response rate 61.1%), new respondents from the SWES 2005 were added. All eligible SWES 2003 and 2005 participants were then followed up in 2010 (wave 3, n = 10,078; response rate 56.8%) and 2012 (wave 4, n = 9,880; response rate 56.7%) as well even if they did not respond in earlier data collections.

Participants in SLOSH are follow-up regardless of whether they have continued to work or not, and hence invited to complete one of the two mail-in self-completion questionnaires: one is addressed to “gainfully employed,” in other words those who had at least 30% full-time paid work; the other one is to “not gainfully employed,” meaning those working less or who are outside of the labor force (e.g., retirees and people on parental or sick leave). The questionnaires include a number of items regarding self-rated health, health behaviors, work environment and social situation. Data from SLOSH are also linked, through the individual social security numbers, to registry data in Statistics Sweden. The study has been approved by the Regional Research Ethics Board in Stockholm.

The current study was based on respondents from SLOSH, wave 4 (2012). In total, 9,880 persons participated, of which 7,325 were gainfully employed and 2,555 not gainfully employed. Subjects with incomplete data on any of the variables of interest were excluded in the analyses, resulting in a final study sample of 8,716 participants. Thus, 11.8% of subjects were missing due to missing data on some measures.

Women, older and married individuals as well as people with high education are generally overrepresented among the respondents to SLOSH. This also applies to the current study population, see demographics, Table 1.

**Table 1:** Demographic characteristics of study population.

Characteristic	Number	Percent
**Age (24–74)**		
20–29	184	2
30–39	1,058	12
40–49	2,005	23
50–59	2,364	27
≥60	3,105	36
**Sex**		
Men	3,913	45
Women	4,803	55
**Socioeconomic status**		
Unskilled employees	1,365	16
Skilled employees	1,346	16
Assistant non-manual employees	1,219	14
Intermediate non-manual employees	2,721	32
Professionals and upper-level executives	1,795	21
Self-employed	67	1
**Education**		
Primary school	896	10
High school	3,775	43
University <3 years	603	7
University >3 years	3,441	40
**Civil status**		
Single	1,780	20
Married/cohabitant	6,915	80

More detailed information about the cohort, response rate and characteristics of responders vs. non-responders has been published elsewhere [36].

The SLOSH data cannot be made fully publicly available due to legal restrictions. We are not allowed to publish the data set underlying our findings, since that would compromise the integrity and privacy of the study participants. For data requests, please contact the SLOSH data manager, Constanze Leineweber, at constanze.leineweber@su.se.

### Questionnaire measures

#### Mind and body exercises

**MBE practice** was measured by asking the participants one single question, “Do you practice any of the following techniques?” The question consists of two parts, one regarding physically directed MBE practice (yoga, Tai Chi, Qi Gong) and one with a mental focus (meditation, mindfulness, relaxation techniques). The five response options ranged from “no” to “daily” [37]. For the analyses, the respondents were divided into three groups: those who practiced MBE “never,” “seldom” (“a few times a year” + “a few times a month”), or “often” (“a few times a week” + “daily”).

#### Demographics

Demographic factors such as sex, age, education and socioeconomic status were included to investigate possible associations between the items and the MBE practice.

#### Health behaviors

**Physical activity** was assessed with one single question, “How much exercise do you get? Include any walking or cycling you do to or from work,” with four possible response alternatives ranging from “never” to “regularly.” The answers were dichotomized into “seldom or never” (“never” + “very little” + “now and then”) and “regularly” (“regularly”).

**Alcohol consumption/problem drinking** was measured with the CAGE (Cut, Annoyed, Guilty, Eye-opener) questionnaire, which is considered a validated screening technique for identifying alcohol abuse. It consists of four yes/no questions concerning different drinking habits [38, 39].

#### Smoking

The participants were asked if they currently smoked and were given three options: 1 = “yes, daily”, 2 = “now and then”, and 3 = “no”. The answers were then dichotomized into “daily” (“yes, daily”) and “seldom or never” (“now and then” + “no”).

#### Subjective well-being

A number of questions regarding stress and stress-related health problems among participants were also included in the analyses. For some single items or scales the original scale was retained to represent severity of certain health complain, whereas some were considered more suitable to dichotomize to represent presence or absence of health complaints.

**Self-rated health** was assessed with a single question that has been widely used for research, “How would you rate your general state of health?” [40, 41]. The respondents answered on a Likert scale, ranging from 1 (“very poor”) to 5 (“very good”). The answers were dichotomized into “poor” (“very poor” + “rather poor” + “neither poor nor good”) and “good” (“rather good” + “very good”).

**Sleeping problems** were assessed with the established measures *sleep disturbances* (reflecting lack of sleep continuity) and *awakening problems* (reflecting feelings of being insufficiently restored) by asking six questions about the respondents’ sleep habits during the last 3 months, using the Karolinska Sleep Questionnaire [42, 43]. The participants were asked if they had any problems with e.g. falling asleep, sleep continuity, early awakening, and unrefreshed sleep and were given six response options: 1 = “never”, 2 = “seldom”, 3 = “sometimes, a few times a month”, 4 = “often, 1–2 times a week), 5 = “mostly, 3–4 times a week” and 6 = “always, 5 times a week or more”. The answers were dichotomized into “no sleeping problems” (if the respondent chose option 1, 2, 3 or 4 on all the questions in the index) and “sleeping problems” (if the respondent chose option 5 or 6 on any of the questions in the index).

#### Pain

The respondents were asked a single question, “To what extent is pain a health concern for you?” The extent of pain was scored on a scale from 1 (“minor problem”) to 5 (“major problem”).

**Depressive symptoms** were measured by the Symptom Checklist-core depression scale (SCL-CD_6_), a six-item scale selected from the Hopkins Symptom Checklist depression subscale. The SCL-CD_6_ has been found to be a psychometrically valid depression scale which covers six core symptoms of depression (feeling blue, feeling no interest in things, feeling lethargy or low in energy, worrying too much about things, blaming yourself for things, feeling everything is an effort). For each item, the respondents were asked, “How much during the past week has that problem troubled you?” They were given five response alternatives, ranging from 0 (“not at all”) to 4 (“extremely”) [44–46]. A sumscore of the six items ranging from 0–24 was used for the analyses.

**Stress** was assessed with three items from the Long Lasting Stress scale [47]. The participants were asked how they felt during the last three months with regard to certain symptoms related to stress (“I have days when I feel wound up all the time”, “I have days when I feel very pressured all the time”, “I have days when I feel stressed all the time”). The response alternatives ranged from 1 (“not at all”) to 4 (“nearly all the time”) and the mean scale (score 1–4) was used for the analyses.

**Life satisfaction** was measured by a single item, asking the respondents to specify how satisfied or dissatisfied they were with life in general on a scale of 1 (“very dissatisfied”) to 7 (“very satisfied”). Thus, a high score indicates high satisfaction.

#### Cognitive complaints

The participants were asked about difficulties during the past three months with four symptoms regarding cognitive abilities (concentration, decision making, memory and the ability to think clearly). The scale, ranging from 1 (“never”) to 5 (“always”), is originally from The Stress Profile Questionnaire [48, 49], and a mean scale (score 1–5) was used for the analyses.

**Emotional exhaustion symptoms** were measured using the emotional exhaustion subscale of the Maslach Burnout Inventory General Survey, which consists of 5 questions regarding the frequency of the participants’ symptoms of burnout. The response alternatives ranged from 1 (“a few times a year or less”) to 6 (“every day”), and a mean scale (score 1–6) was used for the analyses.

#### Illnesses/Diseases

Data concerning certain self-reported diseases were also included.

**Mental disorders** and **disease in back, joints, or muscle** were measured by the single question, “Do you have or have you had any of the following prolonged and/or severe diseases or complaints/illnesses during the last 2 years, and in that case, how did it affect your life?” The participants were given four response options: 1 = “no”, 2 = “yes, but it doesn’t affect my life”, 3 = “yes, it affects my life to some extent”, and 4 = “yes, it affects my life a lot”. The answers were dichotomized into “no” (“no”) and “yes” (”yes, but it doesn´t affect my life” +”yes, it affects my life to some extent” +”yes, it affects my life a lot”).

### Prescription data

Data from SLOSH questionnaires was supplemented with data on purchases of prescription drugs for all respondents included in the study. These data were obtained from the Swedish Prescribed Drug Register, which contains information about prescription drugs dispensed at Swedish pharmacies; the drugs are classified according to the Anatomical Therapeutical Classification (ATC) system.

The ATC groups included in this study were: N06A (antidepressants), N05B (anxiolytics), N05C (hypnotics), M01A (non-steroidal anti-inflammatory drugs), M02A (topical products for joint and muscular pain), N02B (paracetamol, acetylsalicylic acid), and N02C (antimigraine preparations).

ATC groups M01A, M02A, N02B and N02C were included in the same group, referred to as analgesics.

In Sweden, patients can purchase a maximum 90-day supply of continuous use prescription drugs as included in the Swedish Pharmaceutical Benefits Scheme per fill.

All purchases of drugs within the selected ATC groups during the period from 90 days prior, to 90 days after completing the SLOSH questionnaire were included in the analyses.

Based on this information, the participants were classified into two groups depending on whether they had made any purchases of any such drug (regardless of the amount of drug) or not during the period of interest.

### Statistical analyses

Data analyses were performed using STATA software (version 13).

To test if there were differences between groups with high, medium and low intensity MBE practice regarding sex, education, children at home, socioeconomic classification, physical activity, alcohol abuse, smoking, self-rated health, sleep disturbances, awakening problems, diseases, and purchases of prescription drugs, Pearson’s *χ2* test was used. Separate analyses were performed for mental MBE and physical MBE, respectively.

As for age, pain, depressive symptoms, long-lasting stress, life satisfaction, cognitive complaints and emotional exhaustion symptoms, we chose the Kruskal-Wallis test over the one-way ANOVA, since Bartlett’s test showed unequal variances.

### Ethical approval

The study has been approved by the Regional Ethical Review Board in Stockholm (dnr 2013/2173-32). All study participants gave their informed consent, and data were analyzed anonymously.

## Results

The overall participation in MBE among the 8,716 respondents in the study population for meditation/mindfulness/relaxation (mental MBE) techniques was 15% (n = 1,340), while for yoga/Tai Chi/Qi Gong (bodily MBE) it was 9% (n = 783). Regarding the frequency of practice of those who reported practicing mental MBE, 396 persons (30%) did it on a regular basis (several times a week or more often) and 944 persons (70%) more seldom (a few times a month to a few times a year). As for the frequency of practice for those who reported practicing bodily MBE, 189 persons (24%) were doing it on a regular basis and 594 (76%) more seldom.

The results show several statistically significant differences regarding demographic characteristics, health behaviors, self-assessed general health, perceived stress, and drug treatment respectively between people who practice MBE extensively compared to people who do not practice MBE.

One of the most notable results was the use of antidepressants among study participants within the different groups of mental MBE. The analysis shows that study participants, who engage in some form of mental MBE a few times a week or more often, are three times more likely to purchase antidepressants compared to people who never practice mental MBE (17% vs 5%). The findings for anxiolytics were almost identical, and for hypnotics and analgesics similar results were observed, although the differences were somewhat smaller (Table 2).

**Table 2:** 

Purchase of prescribed drugs	MBE practice (meditation/mindfulness/relaxation techniques)			Test of differences
	Never	Seldom	Often	
	N (%)	N (%)	N (%)	
**Analgesics**				**p<0.001**^a^
No	6,410 (87)	809 (86)	309 (78)	
Yes	966 (13)	135 (14)	87 (22)	
**Antidepressants**				**p<0.001**^a^
No	6,973 (95)	828 (88)	328 (83)	
Yes	403 (5)	116 (12)	68 (17)	
**Anxiolytics**				**p<0.001**^a^
No	7,217 (98)	903 (96)	368 (93)	
Yes	159 (2)	41 (4)	28 (7)	
**Hypnotics**				**p<0.001**^a^
No	7,088 (96)	867 (92)	363 (92)	
Yes	288 (4)	77 (8)	33 (8)	

^a^ = *χ*^2^

The analysis also showed significant and positive associations between mental MBE practice and sleeping problems, pain, and mental disorders that were consistent with the pattern for purchases of prescribed drugs mainly for sleep, pain and mental health problems (Table 3). Participants with a regular practice of mental MBE were, for example, three times more likely to suffer from some kind of mental disorder compared to people who never practice mental MBE (13% vs 4%).

**Table 3:** 

	MBE practice (meditation/mindfulness/relaxation techniques)			Test of differences
	Never	Seldom	Often	
	N (%)/Mean±SD	N (%)/Mean±SD	N (%)/Mean±SD	
**Self-rated health**				**p<0.001**^a^
Good	5,875 (80)	718 (76)	280 (71)	
Bad	1,501 (20)	226 (24)	116 (29)	
**Sleep disturbances**				**p<0.001**^a^
No	5,987 (81)	681 (72)	289 (73)	
Yes	1,389 (19)	263 (28)	107 (27)	
**Awakening problems**				**p<0.001**^a^
No	6,195 (84)	723 (77)	302 (76)	
Yes	1,181 (16)	221 (23)	94 (24)	
**Pain**				**p<0.001**^b^
1–5	2.003±1.1683	2.100±1.2454	2.407±1.3922	
**Depressive symptoms**				**p<0.001**^b^
0–24	4.2±4.6	6.0±5.5	5.9±5.7	
**Long-lasting stress**				**p<0.001**^b^
1–4	1.6±0.5	1.8±0.6	1.8±0.7	
**Life satisfaction**				**p = 0.391**^b^
1–7	5.7±1.2	5.6±1.3	5.7±1.4	
**Cognitive complaints**				**p<0.001**^b^
1–5	2.0±0.8	2.3±0.8	2.3±0.9	
**Emotional exhaustion**				**p<0.001**^b^
1–6	2.1±1.1	2.5±1.3	2.6±1.4	
**Mental disorders**				**p<0.001**^a^
No	7,057 (96)	859 (92)	343 (87)	
Yes	260 (4)	79 (8)	51 (13)	
**Back/joint/muscle problems**				**p<0.001**^a^
No	5,218 (71)	629 (67)	245 (62)	
Yes	2,102 (29)	310 (33)	149 (38)	

^a^ = *χ*^2^

^b^ = Kruskal-Wallis

Furthermore, high-intensity mental MBE practice also showed a positive association with low ratings of self-rated health and high scores on the measures of depressive symptoms, long lasting stress, cognitive complaints, emotional exhaustion and back, joint or muscle problems. However, no significant differences regarding satisfaction with life in general were found (Table 3).

Regarding sex, the practice of meditation, mindfulness and relaxation techniques was more common among women; approximately three times more women than men are practicing on a regular basis. Physically active people participate to a greater extent in mental MBE compared with physically inactive. No significant correlation between alcohol consumption and MBE was found, while the relationship between smoking and MBE is more complex (Table 4).

**Table 4:** 

	MBE practice (meditation/mindfulness/relaxation techniques)			Test of differences
	Never	Seldom	Often	
	N (%)/Mean±SD	N (%)/Mean±SD	N (%)/Mean±SD	
**Sex**				**p<0.001**^a^
Men	3,600 (92)	231 (6)	82 (2)	
Women	3,776 (79)	713 (15)	314 (7)	
**Age (years)**				**p<0.001**^b^
24–74	53.5±11.5	51.7±11.0	53.7±10.7	
**Socioeconomic status**				**p<0.001**^a^
Unskilled employees	1,230 (90)	85 (6)	50 (4)	
Skilled employees	1,219 (91)	86 (6)	41 (3)	
Assistant non manual employees	1,017 (83)	143 (12)	59 (5)	
Intermediate non manual employees	2,205 (81)	383 (14)	133 (5)	
Professionals and upper-level executives	1,465 (81)	227 (13)	103 (6)	
Self-employed	63 (94)	4 (6)	0 (0)	
**Education**				**p<0.001**^a^
Primary school	843 (94)	36 (4)	17 (2)	
High school	3,291 (87)	335 (9)	149 (4)	
University <3 years	513 (85)	65 (11)	25 (4)	
University >3 years	2,729 (79)	508 (15)	204 (6)	
**Children at home**				**p = 0.066**^a^
Yes	2,907 (84)	409 (12)	155 (4)	
No	4,469 (85)	535 (10)	241 (5)	
**Physical activity**				**p<0.001**^a^
Seldom/never	4,101 (88)	397 (9)	144 (3)	
Regularly	3,275 (80)	547 (13)	252 (6)	
**Alcohol consumption (problem drinking)**				**p = 0.985**^a^
No	6,897 (85)	884 (11)	370 (4)	
Yes	479 (85)	60 (11)	26 (4)	
**Smoking**				**p = 0.001**^a^
Daily	640 (88)	50 (7)	40 (6)	
Seldom/never	6,736 (84)	894 (11)	356 (4)	

^a^ = *χ*^2^

^b^ = Kruskal-Wallis

These cross-sectional associations were generally stronger for participants in the group practicing mental MBE than for respondents who participate in bodily-directed MBE. For detailed data regarding physical MBE, see S1 Appendix.

## Discussion

This study examined differences regarding topics such as self-rated health, perceived stress and levels of prescription drugs among people with high-intensity MBE practice compared to people who do not practice MBE, in a large population-based cohort.

The results show a significant and positive relationship between MBE and *poor* self-assessed health, *high* levels of stress, and purchases of psychotropic drugs and analgesics. These cross-sectional relationships were generally stronger for mental MBE than for bodily-directed MBE.

The associations between MBE practice and variables such as sleeping problems, perceived stress, pain, depressive symptoms, mental disorders, cognitive complaints, emotional exhaustion and prescription drugs seen in this study are somewhat surprising given that previous studies have shown a positive effect of MBE on stress and stress-related problems (e.g., anxiety, depression, pain and sleep problems) [2, 6–10, 13–16]. Based on these studies, it would rather be natural to expect that people who practice MBE would have lower levels of stress and other symptoms in an epidemiological study like this. However, several other studies—some of which were conducted on people with mental health problems as well as some that included the general population—have confirmed an association between poor self-reported health, high rates of depression or anxiety and the participation in MBE [33–35, 50]. These results are more in line with our findings. Furthermore, since this is a cross-sectional study, it is impossible to establish cause and effect.

The results may partly be explained by the fact that prescribing physicians in Sweden sometimes recommend actions such as, for example, practicing mindfulness as part of the treatment of stress-related problems, exhaustion, depression and anxiety disorders [51]. In addition to such recommendations, patients can find information regarding benefits of both yoga, tai chi, qi gong, meditation, mindfulness and relaxation techniques for stress and stress-related symptoms on “Healthcare Guide 1177,” a Swedish website consisting of information and advice on health and healthcare that builds upon a quality-assured medical database and aims to inform the general public regarding medical concerns [52].

The fact that persons who are on, for example, antidepressants are significantly more likely to engage in some form of MBE compared to people who are not on medication, may also be an indication that persons suffering from conditions such as mental disorders tend to seek help more extensively than others. This interpretation is consistent with Andersen’s Behavioral Model of Health Services Use, an established model for conceptualizing professional help-seeking [53, 54]. Andersen’s model distinguishes three interrelated predicting factors for help-seeking: need factors (perceived as well as evaluated need), predisposing factors (personal, social and cultural factors) and enabling factors (organizational factors that affect the availability and affordability of health care). Using this model as a framework, it might be easier to understand and explain our results. Most likely, people with perceived poor self-rated health, high stress levels and high levels of prescription drugs purchases have a stronger demand for help than others, and thus tend to participate in MBE more extensively. Additionally, Thorne et al. describe the use of complementary and alternative methods/CAM (e.g. MBE) as a way for the patient to engage in and take responsibility for their own health and treatment, and as a *complement* rather than an *alternative* to conventional treatment [55].

Other studies using Andersen’s model have shown that women are more likely than men to seek medical help, and that education and income are also important in the help-seeking process [56]. This is consistent with earlier studies on CAM-use [50] as well as with our findings that gender, SEI and education correlate with participation in MBE.

Despite the association between MBE and indications of poor self-assessed health and stress, there was no significant difference in satisfaction with life in general between the three MBE groups.

### Strengths

To the best of our knowledge, only a few earlier studies have examined the associations between MBE and pharmacological treatment, and none specifically on medication for stress-related symptoms. There is also a lack of population-based cohort studies regarding the prevalence of MBE. In this respect, this study contributes to widening the knowledge about the use of MBE in the general population and its relation to demographics, self-rated health and purchase of prescribed drugs. The SLOSH sample is large and can be considered approximately nationally representative of the Swedish working population, allowing broad generalizability of the results. A strength is also that the study is based on both self-reported and register data. The Swedish Prescribed Drug Register represents one of the largest population-based pharmacoepidemiological databases in the world, and covers all prescription drugs, thus providing robust data for descriptive and analytical studies [57].

### Limitations

The cross-sectional design of the current study does not allow for causal inferences; hence, the observed associations between MBE and use of antidepressants could represent different causal relationships such as, for example, that high-intensity MBE practice leads to high purchase of prescribed antidepressants or (perhaps more likely) that poor self-assessed health leads to a more extensive use of both MBE and prescribed drugs. To further investigate these relationships, longitudinal studies that can account for temporality between covariates and MBE use are needed.

Furthermore, the questions regarding MBE practice are broad and do not distinguish between, for example, relaxation techniques downloaded at home as an audio file or a mindfulness course led by an authorized instructor. For future studies, more specified questions regarding kinds of MBE, duration, and the quality of the MBE activity should be used.

Concerning the assessment of prescription drugs, we have to acknowledge that neither prescription nor purchase of drugs is the same as adherence. Furthermore, not everyone diagnosed with depression is treated with antidepressants, while others use them for other indications (such as premenstrual syndrome). Thus, the relationship between diagnosis, purchase of prescribed drugs and intake of the drugs is not straightforward.

Furthermore, that a relatively large group declined to respond to the survey is not optimal. If the non-response is selective, it might lead to under- or overestimated prevalence of MBE. As people who were originally working are followed up on over time, the non-working population is also underrepresented in the SLOSH sample.

## Conclusion

Overall, the study shows that frequent participation in mind and body exercises is associated with high levels of purchases of psychotropic drugs and analgesics as well as with poor self-assessed health and high levels of stress, most strongly so for mental MBE practices such as meditation and mindfulness. However, since this is a cross-sectional study, it is impossible to establish cause and effect and to further investigate the associations found; longitudinal studies with more advanced statistical methods are needed.

An interesting finding is that, despite the association between MBE and indications such as, for example, poor self-assessed health and stress, there is no significant difference in satisfaction with life in general between the three MBE groups; this is something that would be interesting to investigate further in future studies.

## Supporting information

S1 Appendix Physical MBE. Data on physical MBE.
(DOCX)
