# Supplementary material for: Prevalence of mind and body exercises (MBE) in relation to demographics, self-rated health, and purchases of prescribed psychotropic drugs and analgesics
Source: PLoS One. 2017 Sep 15;12(9):e0184635. doi: 10.1371/journal.pone.0184635 (PMC5600368; doi:10.1371/journal.pone.0184635)
Supplement: S1 Appendix — Data on physical MBE. (DOCX) [file pone.0184635.s001.docx]

Appendix 1. Physical MBE.

| **Purchase of prescribed drugs** | **MBE practice**  **(yoga/Tai Chi/Qi Gong)** | | | **Test of differences** |
| --- | --- | --- | --- | --- |
|  | Never | Seldom | Often |  |
|  | N (%) | N (%) | N (%) |  |
| **Analgesics** | | | | **p=0.296^a^** |
| No | 6,859 (86) | 502 (85) | 167 (88) |  |
| Yes | 1,074 (14) | 92 (15) | 22 (12) |  |
| **Antidepressants** | | | | **p<0.001^a^** |
| No | 7,428 (94) | 528 (89) | 173 (92) |  |
| Yes | 505 (6) | 66 (11) | 16 (8) |  |
| **Anxiolytics** | | | | **p=0.365^a^** |
| No | 7,729 (97) | 578 (97) | 181 (96) |  |
| Yes | 204 (3) | 16 (3) | 8 (4) |  |
| **Hypnotics** | | | | **p<0.001^a^** |
| No | 7,593 (96) | 548 (92) | 177 (94) |  |
| Yes | 340 (4) | 46 (8) | 12 (6) |  |

a=*χ*^2^

Table 1.

|  | **MBE practice**  **(yoga/Tai Chi/Qi Gong)** | | | **Test of differences** |
| --- | --- | --- | --- | --- |
|  | Never | Seldom | Often |  |
|  | N (%)/Mean+SD | N (%)/Mean+SD | N (%)/Mean+SD |  |
| **Self-rated health** | | | | **p=0.607^a^** |
| Good | 6,246 (79) | 478 (80) | 149 (79) |  |
| Bad | 1,687 (21) | 116 (20) | 40 (21) |  |
| **Sleep disturbances** | | | | **p<0.001^a^** |
| No | 6,380 (80) | 436 (73) | 141 (75) |  |
| Yes | 1,553 (20) | 158 (27) | 48 (25) |  |
| **Awakening problems** | | | | **p<0.001^a^** |
| No | 6,628 (84) | 446 (75) | 146 (77) |  |
| Yes | 1,305 (16) | 148 (25) | 43 (23) |  |
| **Pain** | | | | **p=0.935^b^** |
| 1-5 | 2.028+1.1840 | 2.072+1.2522 | 2.074+1.2861 |  |
| **Depressive symptoms** | | | | **p<0.001^b^** |
| 0-24 | 4.4+4.8 | 5.7+5.5 | 4.8+5.3 |  |
| **Long-lasting stress** | | | | **p<0.001^b^** |
| 1-4 | 1.6+0.6 | 1.8+0.6 | 1.7+0.7 |  |
| **Life satisfaction** | | | | **p=0.365^b^** |
| 1-7 | 5.7+1.2 | 5.7+1.3 | 5.7+1.4 |  |
| **Cognitive complaints** | | | | **p<0.001^b^** |
| 1-5 | 2.0+0.8 | 2.3+0.9 | 2.2+0.9 |  |
| **Emotional exhaustion** | | | | **p<0.001^b^** |
| 1-6 | 2.1+1.1 | 2.3+1.3 | 2.3+1.3 |  |
| **Mental disorders** | | | | **p=0.003^a^** |
| No | 7,536 (96) | 548 (93) | 175 (93) |  |
| Yes | 336 (4) | 41 (7) | 13 (7) |  |
| **Back/joint/muscle problems** | | | | **p=0.883^a^** |
| No | 5,540 (70) | 420 (71) | 132 (70) |  |
| Yes | 2,336 (30) | 169 (29) | 56 (30) |  |

a=*χ*^2^

b=Kruskal-Wallis

Table 2.

|  | **MBE practice**  **(yoga/Tai Chi/Qi Gong)** | | | **Test of differences** |
| --- | --- | --- | --- | --- |
|  | Never | Seldom | Often |  |
|  | N (%)/Mean+SD | N (%)/Mean+SD | N (%)/Mean+SD |  |
| **Sex** | | | | **p<0.001^a^** |
| Men | 3,812 (97) | 74 (2) | 27 (1) |  |
| Women | 4,121 (86) | 520 (11) | 162 (3) |  |
| **Age (years)** | | | | **p<0.001^b^** |
| 24-74 | 53.5+11.4 | 50.0+11.3 | 54.6+10.5 |  |
| **Socioeconomic status** | | | | **p<0.001^a^** |
| Unskilled employees | 1,302 (95) | 50 (4) | 13 (1) |  |
| Skilled employees | 1,277 (95) | 47 (3) | 22 (2) |  |
| Assistant non manual employees | 1,100 (90) | 95 (8) | 24 (2) |  |
| Intermediate non manual employees | 2,421 (89) | 227 (8) | 73 (3) |  |
| Professionals and upper-level executives | 1,578 (88) | 165 (9) | 52 (3) |  |
| Self-employed | 66 (99) | 0 (0) | 1 (1) |  |
| **Education** | | | | **p<0.001^a^** |
| Primary school | 864 (96) | 23 (3) | 9 (1) |  |
| High school | 3,532 (94) | 189 (5) | 54 (1) |  |
| University <3 years | 550 (91) | 41 (7) | 12 (2) |  |
| University >3 years | 2,987 (87) | 340 (10) | 114 (3) |  |
| **Children at home** | | | | **p=0.004^a^** |
| Yes | 3,120 (90) | 275 (8) | 76 (2) |  |
| No | 4,813 (92) | 319 (6) | 113 (2) |  |
| **Physical activity** | | | | **p<0.001^a^** |
| Seldom/never | 4,389 (94) | 211 (5) | 42 (1) |  |
| Regularly | 3,544 (87) | 383 (9) | 147 (4) |  |
| **Alcohol consumption (problem drinking)** | | | | **p=0.597^a^** |
| No | 7,414 (91) | 557 (7) | 180 (2) |  |
| Yes | 519 (92) | 37 (6) | 9 (2) |  |
| **Smoking** | | | | **p<0.001^a^** |
| Daily | 695 (95) | 28 (4) | 7 (1) |  |
| Seldom/never | 7,238 (91) | 566 (7) | 182 (2) |  |

a=*χ*^2^

b=Kruskal-Wallis

Table 3.
